# *Skimmia anquetilia* N.P. Taylor and Airy Shaw (Rutaceae): A Critical Appriasal of its Ethnobotanical and Pharmacological Activities

**DOI:** 10.3389/fpls.2022.930687

**Published:** 2022-07-29

**Authors:** Masarat Nabi, Nahida Tabassum, Bashir Ahmad Ganai

**Affiliations:** ^1^Department of Environmental Science, University of Kashmir, Srinagar, Jammu and Kashmir, India; ^2^Department of Pharmaceutical Sciences, University of Kashmir, Srinagar, Jammu and Kashmir, India; ^3^Centre of Research for Development, University of Kashmir, Srinagar, Jammu and Kashmir, India

**Keywords:** Ornamental shrub, Phytochemistry, Pharmacological activities, *Skimmia anquetilia*, Traditional uses

## Abstract

*Skimmia anquetilia* N.P. Taylor and Airy Shaw (Rutaceae) is a perennial, aromatic, gregarious wild ornamental shrub native to the Western Himalaya. The plant is used in the traditional medicinal system to treat copious health conditions like rheumatism, fever, inflammation, headache, influenza, body-ache, clearing of the nose, diabetes, lowering the body temperature, smallpox, wounds, burns, snake, and scorpion bites. Phytochemical and gas chromatography-mass spectrometer (GC-MS) analysis of *S. anquetilia* showed the presence of alkanes, alkenes, carboxylic acids, fatty acids, and their esters, simple coumarins, terpenes, phenylpropanoid, and so on. These active principles exhibit a wide array of pharmacological effects, including anti-inflammatory, antioxidant, anti-cancerous, anti-feedant, and antibacterial properties. Most pharmacological studies were based on the essential oil and the crude extracts of the plant and the bioactive compounds responsible for the bioefficacy have not been well-identified. Further investigations are required to transform the experience-based claims on the use of *S. anquetilia* in traditional medicine practices into evidence-based information. Detailed *in-vitro* and *in-vivo* studies on the mechanisms of action of pure bioactive compounds and more elaborate toxicity studies to ensure plant safety for human use should be conducted. This review recapitulates the current status of its use in the ethnobotany, phytochemistry, and pharmacological activities. It also offers a critical assessment of the plant’s existing information which would help to recuperate its potential as a source for drug development of lead molecules.

## Highlights

- *S. anquetilia* is used as a folklore medicine for rheumatism, inflammation, smallpox, headache, fever, and as an antidote.
- Over 130 bioactive phytoconstituents have been identified from *S. anquetilia*.
- Terpenes, glycosides, and fatty acids were identified as major phytoconstituents.
- Pharmacological studies such as antibacterial, anti-inflammatory, antioxidant, anticancer, anti-arthritic, etc., have been reported.
- *S. anquetilia* is cytotoxic to cell lines such as MCF-7, HeLa, PC-3, and Caco-2.

## Introduction

Medicinal plants have achieved broader recognition in recent times since these plants are natural products, they have minimal side effects and better effectiveness than their synthetic equivalents (Batiha et al., 2020). Approximately 80% of people in the world rely upon conventional medicine as a vital source of their basic medical care (Ekor, 2014). Most treatments use medicinal plant extracts and bioactive molecules (Michel et al., 2020). Medicinal plants are important sources of crude drugs that are used to treat various pathological conditions to maintain a status of well-being (Shakya, 2016). Medicinal plants have always been a potential source to treat various ailments, either in the form of traditional preparations or as pure active principles, and perhaps they are often the only source of medicine for the majority of folks in the developing nations (Taylor et al., 2001).

India has a long history of conventional medicinal systems and a lot of knowledge can be acquired from even a common man about herbal medicines of preventive and therapeutic significance. Out of 17,000–18,000 angiospermic species in India, over 7,000 plant species have been identified with medicinal properties (Kala et al., 2006) and of these, ∼1,748 plant species are being used as medicinal plants in the Himalayan region (Joshi et al., 2016). Medicinal plants have been reported to possess several biological activities such as anticancer (Mlilo and Sibanda, 2022), antimicrobial (Chan et al., 2022), anti-diabetic (Ahmed et al., 2022), anti-inflammatory (Rahmawati et al., 2021; Doe et al., 2022), antiviral (Shahrajabian et al., 2022), anti-feedant (Kerebba et al., 2022), antioxidant (Appiah-Opong et al., 2022), and anti-spasmolytic activities (Batiha et al., 2020; Hussain et al., 2020). These activities have been ascribed to their active principles *viz*: coumarins, glycosides, saponins, flavonoids, steroids, tannins, carotenoids, phenolics, phenols, alkaloids, terpenes, etc. (Nazir et al., 2020; Sarkar, 2020). One of the basic requirements for primary health care success is the accessibility and use of appropriate drugs. Conventional medicine is still the most inexpensive and easily available source of treatment in the primary healthcare system.

*S. anquetilia* is a medicinal plant that belongs to the genus *Skimmia* of the Rutaceae family (Saqib and Sultan, 2005). The plant is endemic to the Western Himalaya and is distributed in the mountain ranges of Afghanistan and Indian sub-continent: India, Pakistan, and Nepal (Nissar et al., 2018). In India, the plant originates in the subalpine region of the Garhwal (Gaur, 1999), Jammu and Kashmir, Uttar Pradesh, and Himachal Pradesh (Walters et al., 1986; Sharma et al., 1993). In the conventional medicinal system, different parts of the plant have been used for the treatment of various ailments in parts of the Western Himalayas of India, Nepal, and Pakistan (Epifano et al., 2015). The purpose of this review is to provide an up-to-date and comprehensive overview of the botany, phytochemistry, traditional uses, and pharmacological activities of *S. anquetilia*. Furthermore, the present knowledge obtained mainly from experimental studies was critically evaluated to provide evidence and validation for local and traditional uses of *S. anquetilia* and to suggest future research scenarios and prospective therapeutic uses for this plant.

## Materials and methods

### Searching strategies

**FIGURE 1:**
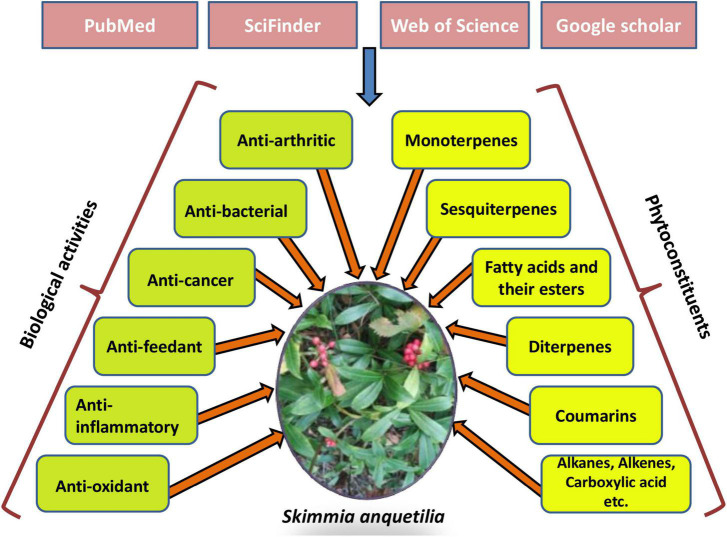
Several databases used to access various phytoconstituents and pharmacological activities of *Skimmia anquetilia*.

An extensive literature search related to the plant species *S. anquetilia* of the genus *Skimmia* was conducted to gather all relevant information about the traditional uses, phytochemicals, and pharmacological activities. Publicly accessible databases and primary sources were searched, including PubMed, SciFinder, Web of Science, Science Direct, Google Scholar, and so on (Figure 1). A large number of literature articles published from 1956 to 2022 were reviewed. The extracted data included vernacular plant names, plant descriptions, traditional uses, purified compounds, and pharmacological activities. The species name was validated using The Plant List (2013).^1^ All studies and reviews that investigated the ability of *S. anquetilia* to heal illnesses in a laboratory (*in-vitro*) and animals (*in-vivo*) were included as long as the effects were explicitly stated.

### Inclusion and exclusion criteria

The literature published up to 2022 was used in this review paper to assess the biological efficacy of *S. anquetilia* concerning six health conditions related to humans. The search was limited to studies published in the English language, independent of the sample size or prospective development period.

### Data extraction

The titles, abstracts, and full articles were used to make a preliminary assessment of the publications by the researchers. Manuscripts that met the study’s predetermined addition and exemption criteria were selected and included. The papers were then used to collect the necessary data on experimental design (animal model and extraction methods), interventions delivered, and treatment findings.

## Collection and identification of plant specimen

The whole plant of *S. anquetilia* N.P. Taylor and Airy Shaw was obtained from the Doodhpathri area (geographical coordinates 74^o^33′35.81″N and 33^o^52′10.87″E) of Budgam district, Kashmir, India at an altitude of 2,814 m above msl. The voucher specimen No. 3152-(KASH) was identified and deposited at the Herbarium, Department of Botany, University of Kashmir. The plant specimen collection did not include endangered or protected species.

## Vernacular names

*S. anquetilia* is known as “Nair” in Garhwal and “Patrang,” “Nar,” “Barru” or “Shalangli” in Punjab, “Nairpatti,” “Nayalpatti” or “Nihar” in Kumaun, “Kasturchara” or “Gurlpatta” in Jaunsar, “Naer Patar,” “Inga,” “Patar,” “Nar,” or “Near” in Kashmir (Goel et al., 1989), and “Kedarpatti” in other hilly areas of India (Negi et al., 2011).

## Plant description

**FIGURE 2:**
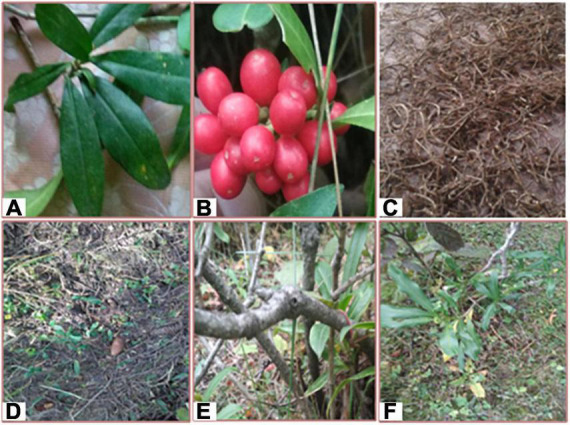
**(A)** Leaves of *Skimmia anquetilia*, **(B)** Berry of *S. anquetilia*, **(C)** Dried root of *S. anquetilia*, **(D)** Plant in nature, **(E)** Stem of *S. anquetilia*, **(F)** Whole plant of *S. anquetilia*.

*S. anquetilia* is an aromatic, perennial, evergreen, gregarious, usually dioeciously, or monoclinous shrub (Gondwal et al., 2018), often cultivated for decorative purposes (Kumar et al., 2012; Figure 2). It has an erect, aerial, cylindrical, faintly fissured, densely branched, glabrous, yellowish, 1.5 m tall, and a 5′ wide stem. Leaves are alternate, simple, often crowded at the tips of branches, petiolate, unicostate, reticulate venation, coriaceous, marginate, oblanceolate, glands with essential oils, narrowly cuneate at base, acute at apex; terminal, paniculate, peduncle; with corymbose inflorescences. The root is tapering, thick, and branched. Flowers are yellowish, complete, indorous, hypogynous, pedicellate, and ebracteate; calyx (4 or 5), connate; petals (5), imbricate; stamens (5), filaments subulate, anthers ellipsoid, with fleshy drupaceous berry fruit, bright red-colored, ovoid (Nissar et al., 2016, 2021).

## Ethnobotany of *S. anquetilia*

Ethnobotany is a field of research that applies indigenous plant knowledge to health care. In India *S. anquetilia* has long been used in Ayurveda and Unani system of medicine, which describes its numerous uses (Table 1). The paste prepared from the roots of *S. anquetilia* has been used as an antidote against scorpion and snake bites (Bhattarai, 1992; Ahmed et al., 2004; Qureshi et al., 2009; Gondwal et al., 2012b). The powder of the plant bark has been found effective in curing wounds and burns (Negi et al., 2011; Singh and Rawat, 2011). The cold infusion of fresh leaves has been used to treats smallpox (Namiki, 1990; Saqib and Sultan, 2005; Prakash et al., 2011; Kumar et al., 2012), headache, and fever (Bhattarai, 1992; Gaur et al., 1993). The smoke of dried leaves has been used for freshness (Bhattarai, 1992; Gaur et al., 1993; Kunwar et al., 2010), and air purification (Chopra et al., 1956; Chauhan et al., 2017). Some tribal sections of India use its leaves in the preparation of curries (Anon, 1966; Chauhan et al., 2017), an alcoholic drink *“Soor”* high in calories (Rana et al., 2004), and as a flavoring agent (Anon, 1966; Chauhan et al., 2017). A paste prepared with the mixture of leaves of *S. anquetilia* and turmeric has been used to treat inflammation and rheumatism (Negi et al., 2011; Singh and Rawat, 2011). In Nepal, leaf infusion is taken for headache and for freshness, leaves are aromatic and used for headache and general fever (Baral and Kurmi, 2006). Personal communications with locals highlighted that that the leaf extract is used to treat diabetes in the Kupwara district of Kashmir valley. Moreover, its dried leaf powder has been used as an insecticidal and pesticidal agent (Bhattarai, 1992). The whole plant has been used in anesthesia and to treat several other health complications like pneumonia, paralysis, and lung cancer (Wani et al., 2016).

**TABLE 1:** Traditional uses of *Skimmia anquetilia*.

Place	Part used	Mode of use	Traditional use	References
NA	Dried leaves	Smoke	To purify air	Chopra et al., 1956
Some tribal hilly areas of Himalaya	Fresh leaves	NA	Used in curries and as flavoring agent	Anon, 1966
NA	Dried leaves	Powder	Used as pesticide, insecticide	Bhattarai, 1992
NA	Fresh leaves	Infusion	Treatment of fever, freshness, headache, smallpox	Bhattarai, 1992
Nepal	Dried leaves and flowers	Smoke	Burned leaves and flowers are used for air purification and keep off evil spirits in the intention of accelerating the patient’s healing.	Bhattarai, 1992
Tons valley of Gaharwal Himalaya	Leaves	NA	Energy rich alcoholic drink known as “Soor” is prepared	Rana et al., 2004
Far-Western Nepal	Leaves	Infusion	Used for freshness and to treat headache	Kunwar et al., 2010
NA	Bark	Powder	To heal burns and wounds	Negi et al., 2011; Singh and Rawat, 2011
Rawain valley of Utarkashi, Uttarakhand	Leaves	Paste	A mixture of fresh leave and turmeric is used to treat rheumatism and inflammation	Negi et al., 2011; Singh and Rawat, 2011
NA	Root	Paste	Used to treat scorpion, snake bites	Gondwal et al., 2012b
Pir-Panjal Range of Himalayas	Whole plant	NA	Paralysis, pneumonia, lung cancer, anesthesia	Wani et al., 2016

NA, not available.

## Phytochemistry

The isolation and identification of secondary metabolites are vital to discover novel drugs to treat diseases. Several studies have been conducted on the phytoconstituents of *S. anquetilia* (Epifano et al., 2015). Over 130 compounds such as fatty acids and their esters, alkanes, alkenes, carboxylic acids, terpenes (such as monoterpenes, diterpenes, sesquiterpenes), phenylpropanoids, etc., have been identified using the GC-MS technique. Most of the phytochemicals have been identified from the essential oils of different parts of *S. anquetilia*. For instance, seventy compounds have been identified using the GC-MS technique from the essential oils of fruit pulp and seeds of *S. anquetilia* (Prakash et al., 2011). Common compounds found in both essential oils were fatty acids and their esters, whereas compounds such as α-cadinol, α-terpineol, selinene, neo-isolongifolene, linalool, *cis*-Z-α-bisabolene oxide, and aromadendrene were found to be the main difference among them (Prakash et al., 2011). Furthermore, more than fifty compounds have been identified from the essential oils of flowers and leaves of *S. anquetilia* and the majority of compounds were found to be monoterpenes and sesquiterpenes such as β-phellandrene (18.4, 1.8%), geijerene (15.0, 2.0%), germacrene B (2.0, 11.6%), linalyl acetate (11.2, 7.3%), linalool (9.4, 9.5%), α-terpineol (4.4, 5.6%), and pregeijerene (5.6, 0.2%) (Gondwal et al., 2012b). To date, only six glycosides (simple coumarins) namely, skimminan (Sharma et al., 2008a), ulopterol, skimmin, osthol, esculetin, and scopoletin (Sharma et al., 2008a,b) have been isolated through column chromatography and identified *via* nuclear magnetic resonance (NMR), correlation spectroscopy (COSY), heteronuclear multiple quantum coherence (HMQC), heteronuclear multiple bond correlation (HMBC), and nuclear overhauser effect spectroscopy (NOESY) techniques from the methanolic leaf extract of *S. anquetilia*. The identified phytoconstituents of *S. anquetilia* are presented in Table 2.

**TABLE 2:** Significant bioactive compounds isolated from *Skimmia anquetilia*.

Class	Bioactive compound(s)	Plant part(s)	References
Alkanes (Straight-chain)	Tetracosane, nonacosane, docosane, heneicosane, heptacosane	Seeds and fruit pulp	Prakash et al., 2011
Alkene	1-Octadecene	Seeds	Prakash et al., 2011
Benzoic acid esters	Dibutyl phthalate	Fruit pulp	Prakash et al., 2011
Carboxylic acid	Ethylhexanoic acid	Seeds	Prakash et al., 2011
Carboximidic acid	Octadecanamide	Fruit pulp	Prakash et al., 2011
Cyclohexenones	Cryptone	Leaves, seeds and fruit pulp	Prakash et al., 2011; Chauhan et al., 2017
Fatty acids and esters of fatty acids	Neryl acetate, octadecanoic acid, tridecanoic acid, oleic acid, *n*-hexadecanoic acid, methyl oleate, methyl linolenate, hexadecanoic acid,1,1-dimethylethyl ester, hexadecanoic acid, methyl ester, dodecanoic acid, hexadecanoic acid, butyl ester, heptadecanoic acid, tetradecanoic acid, geranyl acetate	Leaves, seeds, Stem bark, and root bark	Mathela et al., 1992; Prakash et al., 2011; Gondwal et al., 2012b; Wani et al., 2016; Chauhan et al., 2017
Glycosides	Skimminan {7,8-dihdroxy-6-[3′- β-D-glucopyranosyloxy-2′(ξ)-hydroxy-3′ methylbutyl]-coumarin}, Skimmin (7-O-β-D-glucopyranosylumbelliferon), osthol [7-methoxy-8-(3-methylbut-2-enyl)-2-chromenone], esculetin (6,7-dihydroxy-chromen-2-one), scopoletin (7-hydroxy-6-methoxy-2H-1-benzopyran-2-one)	Leaves	Sharma et al., 2008a
Phenylpropanoid	Asarone	Seeds and fruit pulp	Prakash et al., 2011
Monoterpenes	β-phellandrene, linalool, myrcene, α-terpineol, geraniol, linalyl acetate, sabinene, β-myrcene, nerol, (*S*)-(+)-carvone acetate, 1,8-cineole, α-phellandrene, α-pinene, terpinen-4-ol, p-cymene, β-pinene, terpinolene, *Z*-β-ocimene, *E*-β-ocimene, α-thujene, camphene, terpinen-4-ol, γ–terpinene, linalyl propionate, D-limonene, terpinyl acetate, Limonene, citral, α-terpinyl acetate, cuminic alcohol, cumaldehyde, (+)-4-carene, β-fenchol, *Cis*-geraniol, *Cis*-ocimene, *Trans*-geraniol	Leaves, flower, stem bark, root bark, seeds, and fruit pulp	Sharma et al., 1966; Sarin, 1977; Gulati, 1982; Goel et al., 1989; Mathela et al., 1992; Prakash et al., 2011; Gondwal et al., 2012b; Wani et al., 2016; Chauhan et al., 2017
Diterpene	Thunbergene, phytol	Leaves, stem bark, and root bark	Prakash et al., 2011; Wani et al., 2016
Sesquiterpenes	Pregeijerene, elemol, dictamnol, α-humulene, pregeijerene B, geijerene, germacrene D, (E,E)-farnesyl acetate, epi-cubebol, δ-cadinene, α-cadinene, γ-elemene, *Cis*-nerolidol, germacrene B, nerolidol, (E)-nerolidol, β-longipinene, (+)-ledene, β-caryophyllene, β-gurjunene, γ-eudesmol, epi-α-muurolol, β-eudesmol, selin-11-en-4- α–ol, α-cadinol, bulnesol, α-bisabolol, E-farnesol, E-farnesyl acetate, α-farnesene, selinene, β-humulene, β-elemene, α-santalol, viridiflorol, vetiverol, longifolenaldehyde, caryophyllene oxide, cedrenol, ledol, (-)-spathulenol, aromadendrene, (+)-farnesol, nerolidyl acetate	Leaves, flower, stem bark, and root bark	Mathela et al., 1992; Prakash et al., 2011; Gondwal et al., 2012b; Wani et al., 2016; Chauhan et al., 2017
Other compounds	Isogeijerene C, dehydrosabina ketone, C_12_H_18_ isomer, 8-epi-dictamnol, < *N*-methyl > methyl anthranilate, 10-epi- γ-eudesmol, bicyclovetivenol, tricyclo [4.4.1.1 (3,8)] dodeca-4,9-diene, *trans*-Z-α-bisabolene epoxide, pyrethrone, longipinane (E)-, neo-isolongifolene, dimethylethyl ester, diepicedrene-1-oxide, didecenyl succinic anhydride, cyclofenchene, *Cis*-Z-α-bisabolene epoxide, *Cis*-linaloloxide, 1-(4-butoxy-2,6 dimethylphenyl) ethanone, dipropyl phthalate, 2,3-dichlorobi phenyl, *Trans*-2,4-decadienol	Leaves and flower	Gondwal et al., 2012b; Chauhan et al., 2017

## Pharmacology of *S. anquetilia*

The varied traditional uses of *S. anquetilia* have contributed to the initiation of several pharmacological studies. Preceding research shows that the *S. anquetilia* extracts exhibit a wide array of bioactivities, *viz*: antibacterial (Sharma et al., 2008a), antioxidant, anti-inflammatory, and other activities like anti-feedant and anticancer activities (Gondwal et al., 2012a; Kumar et al., 2012; Negi et al., 2012; Wani et al., 2016; Table 3). At the same time, a wide array of *in-vivo* and *in-vitro* models has been used to evaluate the pharmacological properties of *S. anquetilia*. Evidence-based laboratory analysis indicates that petroleum ether, chloroform, ethyl-acetate, methanolic, and aqueous extracts of *S. anquetilia* possess several promising pharmacological properties.

**TABLE 3:** Summary of bioefficacy of *Skimmia anquetilia*.

Biological efficacy	Plant part(s) evaluated	Test system	Tested substance	References
Anti-arthritic	Leaves	*In-vitro*	Ethyl-acetate extract	Verma et al., 2020
Antibacterial	Leaves	*In-vitro*	Methanolic extract and active compounds isolated; Skimminan, Skimmin	Sharma et al., 2008a
	Leaves	*In-vitro*	Methanol extract	Nabi et al., 2022a
	Root	*In-vitro*	*n*-Hexane, ethyl-acetate, and methanol extract	Nabi et al., 2022b
Anticancer	Leaves/stem bark/root bark	*In-vitro*	Essential oil	Wani et al., 2016
Anti-feedant	-	*In-vitro*	-	Negi et al., 2012
	Flowers, leaves	*In-vitro*	Essential oil	Gondwal et al., 2012a
Anti-inflammatory	Leaves	*In-vivo*	Petroleum ether, chloroform, ethyl-acetate, methanol, and aqueous extract	Kumar et al., 2012
	Leaves	*In-vitro*	Petroleum ether, chloroform, ethyl-acetate, methanol, and aqueous extract	Kumar et al., 2012
	Leaves	*In-vitro*	Ethyl-acetate extract	Verma et al., 2020
Antioxidant	Seeds and fruit pulp	*In-vitro*	Aqueous extract	Prakash et al., 2011
	Leaves/flowers	*In-vitro*	Aqueous extract and essential oil	Gondwal et al., 2012b
	Leaves	*In-vitro*	*n*-Hexane, dichloromethane, ethyl-acetate, butanol, methanol, and aqueous fractions	John et al., 2014

### Antibacterial activity

The methanol leaf extract and isolated active constituents, namely; skimminan and skimmin of *S. anquetilia* exhibited broad-spectrum antibacterial activity by inhibition of *Agrobacterium tumifaciens, Pseudomonas syringae*, and *Pactobacterium carotovorum* (Gram-negative plant pathogens) at a dose of 200 μg/disc using disc diffusion method. Results showed that the methanol extract and skimminan exhibited inhibitory activities against all three pathogens, whereas skimmin was only effective against *A. tumifaciens.* The highest zone of inhibition (12.6 ± 0.8) was exhibited by methanol extract against *A. tumifaciens* (Sharma et al., 2008a). Recently, (Nabi et al., 2022a,b) reported the antibacterial activity of methanol leaf extract and *n*-hexane, ethyl-acetate, and methanol root extracts of *S. anquetilia* against *Pseudomonas aeruginosa, Escherichia coli, Klebsiella pneumoniae, Salmonella typhi*, and *Staphylococcus aureus* at different concentrations (10, 20, 40, 80, and 160 mg/ml) using the agar well diffusion assay. The methanolic leaf extract showed the highest zone of inhibition against *E. coli* (19.0 ± 0.57), followed by *P. aeruginosa* (18.0 ± 0.57) and *K. Pneumoniae* (17.0 ± 0.57) at 160 mg/ml. Among the root extracts, ethyl acetate extract showed the highest zone of inhibition against *P. aeruginosa* (18.0 ± 1.0), followed by *S. aureus* (17.0 ± 1.0). Furthermore, the minimum inhibitory concentration (MIC) of methanol leaf extract against *P. aeruginosa* (2 mg/ml) and ethyl acetate root extract against *S. aureus* (4 mg/ml) demonstrated therapeutically significant antibacterial activity.

### Anticancer activity

Among the 35,000 plant species screened against cancer, about 3,000 have demonstrated potential anticancer activities (Surendran et al., 2021). Wani et al. (2016) determined the anticancer activity of the essential oils extracted from the leaf, stem bark, and root bark of *S. anquetilia.* The study was carried out on four different cell lines *viz*: MCF-7 (Breast), HeLa (Cervix), PC-3 (Prostate), and Caco-2 (Colon) using sulphorhodamine (SRB) assay. The stem bark essential oil was found to be the most active against all tested human cancer cell lines with IC50 values ranging from 2.71 to 6.21 μg/ml. Leaf essential oil (IC50 3.01 to 114.50 μg/ml) and root bark essential oil (IC50 14.88 to 49.04 μg/ml) exhibited cytotoxic activity against tested human cancer cell lines.

### Anti-inflammatory activity

Although various anti-inflammatory drugs have been discovered and are in clinical use, the inflammation condition is still challenging. Most of the existing drugs are opioids and non-steroidal anti-inflammatory drugs (NSAIDs), but they produce many side effects. Hence, the discovery of novel drugs is necessary. Plants possess various phytoconstituents that have displayed anti-inflammatory properties with few side effects (Virshette et al., 2019). Phytoconstituents, for instance, tannins, saponins, alkaloids, flavonoids, and phytosterols, have shown promising anti-inflammatory activities (Abdul-Nasir-Deen et al., 2020). The anti-inflammatory effect of *S. anquetilia* has been reported previously. Kumar et al. (2012) evaluated the anti-inflammatory activity of *S. anquetilia* leaf extract (LESA) by *in-vitro* and *in-vivo* methods using the human red blood cell (HRBC) membrane stabilization model and the carrageenan-induced rat paw edema model. The authors indicated that the anti-inflammatory effects of LESA revealed the concentration-dependent activity. For HRBC membrane stabilizing agent, *S. anquetilia* methanol extract exhibited the highest anti-inflammatory effect compared to the other leaf extracts and showed a result value of 68.50 ± 1.57. The chloroform, ethyl-acetate, and methanol leaf extract of *S. anquetilia* at a dose of 400 mg/kg showed 58.22, 60.17, and 67.53% inhibition respectively in albino rats. The methanol extract showed a maximum anti-inflammatory activity of 67.53% at a 400 mg/kg dose against the standard drug Diclofenac (10 mg/kg) (Kumar et al., 2012). Recently, Verma et al. (2020) reported the anti-inflammatory activity of ethyl-acetate leaf extract (EESA) of *S. anquetilia* by *in-vitro* methods using the HRBC membrane stabilization model at doses of 50, 100, 200, and 400 mg/ml. The EESA extract exhibited concentration-dependent inhibition, and the maximum inhibitory effect found was 90.70% at 400 mg/ml in comparison with the standard drug diclofenac sodium which showed 94.88% protection.

### Antioxidant activity

Prakash et al. (2011) have analyzed 2,2-diphenyl-1 picryalhydrazide (DPPH) radical scavenging activity, reducing power assay, and chelating activity of Fe^2+^ of aqueous extracts of seeds and fruit pulp of *S. anquetilia* using butylated hydroxytoluene (BHT), catechin, and gallic acid as standards. The results of the study revealed that both extracts exhibited moderate *in-vitro* antioxidant potential. Gondwal et al. (2012b) determined the antioxidant efficiency of essential oils of leaves and flowers of *S. anquetilia* by reducing power, chelating properties of Fe^+2^, and 2′2′-diphenylpicrylhadrazyl (DPPH) radical-scavenging assay. DPPH radical scavenging activity was higher in the leaf essential oil and extract, whereas the maximum chelating activity was observed in the flower’s essential oil and aqueous extract and the highest reducing power was shown by flower essential oil and leaf extract. John et al. (2014) have determined the antioxidant activity of crude methanol extract, *n*-hexane, dichloromethane, ethyl-acetate, *n*-butanol, and aqueous fractions of *S. anquetilia* leaves by eight distinct assays *viz*: 2,2’-azinobis-(3-ethylbenzothiazoline-6-sulpohonic acid) (ABTS) radical cation scavenging activity, the ferric reducing antioxidant power (FRAP), 2,2-diphenyl-1-picrylhydrazyl (DPPH) radical scavenging activity, total phenolic contents (TPC), total flavonoid contents (TFC), total antioxidant activity by phosphomolybdenum method, superoxide anion radical scavenging activity, and metal chelating activity. They opined that the ethyl-acetate fraction showed the highest total phenolic content, 2,2′-azinobis-(3-ethylbenzothiazoline-6-sulpohonic acid) (ABTS) radical cation scavenging activity, the ferric reducing antioxidant power (FRAP), and the DPPH radical scavenging activity. Dichloromethane fraction showed the highest antioxidant activity. The highest superoxide anion radical scavenging activity was displayed by the aqueous fraction. The crude methanolic extract exhibited the highest total flavonoid contents.

### Anti-feedant activity

Anti-feedants are substances with anti-feedant characteristics that, at low concentrations, act on the pest’s extremely specific receptor cells. Anti-feedant sensory-linked neurons either dissuade or inhibit insect feeding (feeding suppressant effect), or limit the functionality of a feeding stimulant receptor of herbivores, or the capacity to attach directly to normal feeding cues like carbohydrates and amino acids (Purrington, 2003). The essential oils from leaves and flowers of *S. anquetilia* showed suppression in the potential of egg-laying by *Caryedon serratus*, damaging the beetle for groundnut seeds at a 1.5% concentration. The suppression increased with the increase in oil concentration with no interference with the further development of larvae in adults (Gondwal et al., 2012a). The same effects of *S. anquetilia* extracts on Lepidoptera (forest pests) have been reported by Negi et al. (2012).

### Anti-arthritic activity

The production of autoantigens in certain arthritic diseases may be due to the denaturation of protein and membrane lysis action. Denaturation of proteins causes the production of autoantigens in conditions such as rheumatic arthritis, cancer, and diabetes, which are considered inflammatory conditions. Therefore, by inhibition of protein denaturation, inflammatory activity can be inhibited. The anti-arthritic activity of ethyl-acetate leaf extract (EESA) of *S. anquetilia* at concentrations 50, 100, 200, and 400 mg/ml was determined using the protein denaturation assay. The results were compared with standard acetylsalicylic acid (100 mg/ml). The EESA extract showed dose-dependent inhibition of protein denaturation, the maximum inhibition of protein denaturation was found 92.41% at 400 mg/ml in comparison to the standard which showed 96.21% inhibition at 100 mg/ml (Verma et al., 2020).

## Future prospects

*S. anquetilia* is proving to be an unreliable option for the future. Various active compounds *viz*, alkanes, alkenes, coumarins, carboxylic acids, fatty acids, and esters of fatty acids, terpenes (monoterpenes, diterpenes, sesquiterpenes), etc., have been reported to be the major bioactive compounds in this plant (Bhatt et al., 2021). The varied bioactivities including anti-arthritic (Verma et al., 2020), anticancer (Wani et al., 2016), anti-inflammatory, antibacterial (Nabi et al., 2022a,b), antioxidant, and anti-feedant (Gondwal et al., 2015) activities have been studied with potential findings. Despite the positive outcome, most of the studies are based on the *in-vitro* models and mechanisms of action are not well-studied. Various traditional medicinal sources indicate that this plant has been used to treat diabetes, smallpox, burn injuries, etc., but, no pharmacological studies have been conducted to validate these activities. Furthermore, in some areas, there is inadequate information and limited research is available. Therefore, further studies concerning the basic chemical composition of phytoconstituents and the mechanisms involved in traditional uses are needed. The pharmacological activities must be experimented to the next levels for generation of novel drugs. This might prove helpful to use its immense therapeutic efficacy as a potent phytomedicine. Thus, systemic research experiments must be carried out for the development of drugs and medicines for their better economic and therapeutic utilization.

## Conclusion

*S. anquetilia* is probably a possible herbal treatment for various diseases. The plant provides several promising perspectives for both traditional as well as modern medicine. *S. anquetilia* is a wealthy source of essential oils containing various important bioactive compounds but there is inadequate information concerning the basic chemical composition and their mechanisms involved. Most of the plant parts have been used in traditional medicine including leaves, stem, flower, fruit, and root bark. Therefore, determining research analysis of the bioactive constituents is needed, particularly its pharmacological properties and toxicity in terms of both *in-vitro* as well as *in-vivo* test systems to authenticate the safety of such plant-based phytochemicals and to develop standard novel drugs.

## Author contributions

MN: conceptualization and writing—review and editing the manuscript. NT and BAG: reviewing and editing the manuscript. All authors contributed to the article and approved the submitted version.

## Footnotes

1
www.theplantlist.org

## Abbreviations

GC-MS: gas chromatography-mass spectroscopy
NMR: nuclear magnetic resonance
COSY: correlation spectroscopy
HMQC: heteronuclear multiple quantum coherence
HMBC: heteronuclear multiple bond correlation
NOESY: nuclear overhauser effect spectroscopy
HRBC: human red blood cell
SRB: sulphorhodamine
NSAIDs: non-steroidal anti-inflammatory drugs
DPPH: 2,2-diphenyl-1-picrylhydrazyl
BHT: butylated hydroxytoluene
ABTS: 2,2′-azinobis-(3-ethylbenzothiazoline-6-sulpohonic acid)
FRAP: ferric reducing antioxidant power
TPC: total phenolic contents
TFC: total flavonoid contents
MIC: minimum inhibitory concentration.
